# Bisphosphonate-related osteonecrosis of jaw (BRONJ): diagnostic criteria and possible pathogenic mechanisms of an unexpected anti-angiogenic side effect

**DOI:** 10.1186/2045-824X-5-1

**Published:** 2013-01-14

**Authors:** Dileep Sharma, Saso Ivanovski, Mark Slevin, Stephen Hamlet, Tudor S Pop, Klara Brinzaniuc, Eugen B Petcu, Rodica I Miroiu

**Affiliations:** 1 Griffith School of Dentistry and Oral Health, Gold Coast Campus, Griffith University, Gold Coast, Queensland, 4222, Australia; 2 School of Healthcare Science, Manchester Metropolitan University, Manchester, United, Kingdom; 3 Department of Orthopaedics, University of Medicine and Pharmacy Targu Mures, Targu Mures, 540000, Romania; 4 Department of Anatomy and Doctoral School, University of Medicine and Pharmacy Targu Mures, Targu Mures, 540000, Romania; 5 Griffith University School of Medicine, Gold Coast Campus, Griffith University, Gold Coast, Queensland, 4222, Australia

## Abstract

Recently, bisphosphonates (BPs) have been widely used in medical practice as anti-resorptive agents owing to their anti-osteoclatic action. In addition, these compounds are also used for their analgesic action and their potential anti-tumour effect. Patients treated with BPs may subsequently develop osteonecrosis of the jaw or maxillary bone after minor local trauma including dental work, recently labelled as bisphosphonate osteonecrosis of jaw (BRONJ). However, the etiopathogenic mechanisms of this pathological condition are poorly understood. Although, several pathways have been proposed for BRONJ occurrence, no single model can explain all morphological changes observed at the macro- and microscopic level. Recent research suggests that BPs may promote an anti-angiogenic effect which contributes directly to the clinical features associated with BRONJ. Remarkably, the anti-angiogenic effect promoting BRONJ might be in keeping with the anti-neoplastic action of BPs. The current review, presents clinical diagnostic criteria. In addition, based on our own experience we describe the histopathological criteria for diagnosis of BRONJ and the possible pathways which may lead to this frustrating pathological condition.

## Introduction

Bisphosphonates (BPs) are a group of pharmacological agents used as anti-osteoclastic, anti- resorptive agents in calcium metabolism disorders such as osteoporosis, multiple myeloma, Paget’s disease and hypercalcemia of malignancy [1]. The primary objectives of administering these drugs are to improve bone morphology, prevent bone destruction and pathologic fractures, and reduce pain associated with the metastatic bone disease whilst decelerating bone resorption [2-4]. Interestingly, BPs resist hydrolysis in the gut and possess an anti-resorptive action inhibiting hydroxyapatite dissolution [5,6]. Licata et al. (2005) and Michaelson et al. (2005) have noted that the most important effect of these pharmacological agents is represented by promoting apoptosis in osteoclasts [7,8].

Chemically, BPs represent pyrophosphate analogs possessing two variable regions, R_1_ and R_2_ on the carbon atom of BPs molecule attached to basic P-C-P structure. This allows variations in molecular structure and a range of potency corresponding to the changes in the structure [9]. The group occupying R_1_ position, usually hydroxyl, enhances the molecule’s affinity to bone (calcium crystals) and the variable group at R_2_ position decides its anti-resorptive action, specifically its potency and efficacy [9].

Classically, BPs have been classified into: non-nitrogen containing BPs (NNBP) and nitrogen containing BPs (NBP) depending on the presence or absence of nitrogen in their R_2_ group. However, Russell et al. [10] further divided the NBPs into Alkyl-amino and Heterocyclic NBPs based on the mode of action (Table 1).

**Table 1:** Bisphosphonates: types and mode of action

**Generation**	**Type**	**Examples**	**Mode of action**
**First**	NNBPs	Etidronate	Formation of an ATP derivative that impairs osteoclast function and induces osteoclastic apoptosis
		Clodronate	
		Tiludronate	
**Second**	Alkyl-amino NBPs	Pamidronate	Inhibits sterol synthesis via the mevalonate pathway specifically inhibiting its Farnesyl pyrophosphate synthase (FPPS) enzyme
		Alendronate	
		Ibandronate	
		Olpadronate	
**Third**	Heterocyclic NBPs	Risedronate	Inhibits FPPS enzyme and stabilize conformational changes
		Zoledronate	

Clinically, both the NNBPs and NBPs are used as antiresorptive agents (Table 2) but the NNBP are known to be less potent and thus are mainly used in management of osteoporosis where as the heterocyclic NBPs are the most potent BPs used in severe bone resorption cases like in malignancies (Table 2).

**Table 2:** Bisphosphonates: potency, administration and main indications

**Type of BPs**	**Potency**	**Administration**	**Main Indications**
**NNBPs**			
Etidronate	1	Oral	Osteoporosis, Paget’s disease of bone
Clodronate	10	Oral/ Intravenous	Osteoporosis, Paget’s disease of bone
Tiludronate	10	Oral	Paget’s disease of bone
**NBPs**			
Pamidronate	100	Intravenous	Osteolytic bone metastases of breast cancer and osteolytic lesions of multiple myeloma, Paget’s disease of bone
Alendonate	500	Oral	Osteoporosis, Paget's disease of bone
Ibandronate	1000	Oral, Intravenous	Osteoporosis
Risedronate	2000	Oral, Intravenous	Osteoporosis, Paget’s disease of bone, osteolytic lesions of multiple myeloma, hypercalcemia of malignancy
Zoledronate	10000	Intravenous	Osteolytic lesions of multiple myeloma and metastases from solid tumors, hypercalcemia of malignancy

### BPs: side-effects

Orally administered BPs may induce recurrent ulcers with burning sensation and blisters in the oral cavity, erosive oesophagitis, oesophagal stenosis, uveitis, gastric ulcerations and abdominal pain [11-13]. However, more serious effects such as bisphosphonate-related osteonecrosis of jaw (BRONJ), is seen most commonly after intravenous NBPs such as pamidronate and zoledronate [14].

### BRONJ: epidemiological trends

The first clinical description of BRONJ by Marx et al. (2003) ushered several reports from all across the world where BPs are used [15]. In general, intravenous nitrogen containing BPs shows higher incidence of BRONJ but a large variation ranging from 0.0% to 28% has been reported [16-18] depending upon the specific type of BPs used, single or multiple BPs used concomitantly or sequentially, duration of therapy and the condition for which BPs were administered. In addition oral BPs have also been associated with BRONJ, although at much lower percentage, not more that 4% [19,20].

### BRONJ: clinical diagnostic criteria

In 2007, the American Association of Oral and Maxillofacial Surgeons and the American Society for Bone and Mineral Research described BRONJ as an area of exposed bone in the maxillofacial region in a patient on BPs or who has taken in the past these pharmacological agents, without any history of radiotherapy. Furthermore, the lesions must not be healed within 8 weeks after identification by a healthcare practitioner to qualify as a BRONJ case [21,22]. Recently, it was reported a clinical variant of BRONJ that lacked bone exposure, albeit fulfilling all other essential criteria for BRONJ [23,24]. This warrants the addition of various other clinical signs and symptoms into the diagnostic criteria (Table 3).

**Table 3:** BRONJ: clinical signs and symptoms

**Classical sign of BRONJ**	**Possible clinical signs and symptoms associated but not limited to BRONJ**
Exposed necrotic jaw bone	Pain in tooth or bone
	Suppuration
	Swelling
	Sinus and fistula related to jaw bone
	Mobility of teeth
	Trismus
	Non-healing extraction sockets
	Soft tissue ulcerations
	Gross mandibular deformity
	Sequestration of bone

A careful application of these criteria is of paramount importance as other intraoral conditions such as gingivitis and periodontal disease, mucositis, periapical lesions due to pulpal infection and resulting osteomyelitis, sinusitis, bone tumours (primary and metastasic) and osteoradionecrosis need to be ruled out.

### BRONJ- histopathological diagnostic criteria

**Figure 1:**
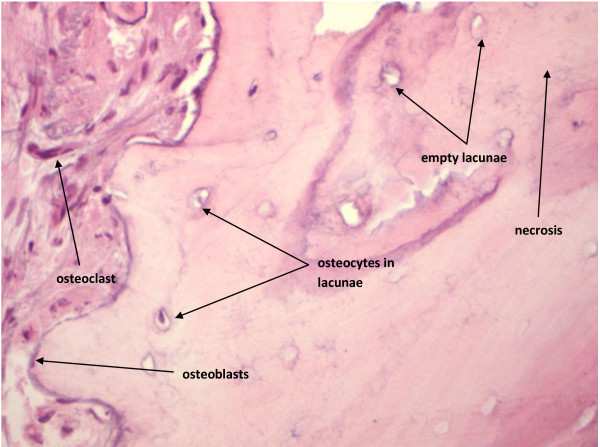
BRONJ: Empty lacunae are seen in the centre of the bone while towards the surface some lacunae display their osteocytes

As other types of osteonecrosis determined by different etiologies, BRONJ is characterized by the presence of osteocyte-depleted bone lacunae. Our diagnostic experience suggests that this avascular necrosis is characterized by lacunae osteocyte depletion which is more obvious in the deeper layers of the bone while the lacunae located towards the surface of the bone lamellae will lose the osteocytes at a later stage (Figure 1).

**Figure 2:**
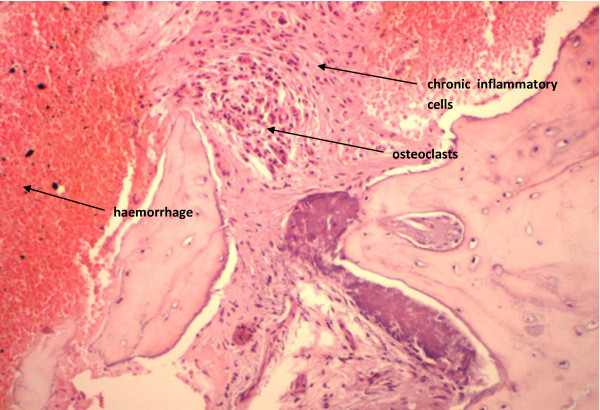
BRONJ: Haemorrhage and chronic inflammatory cells infiltrate

**Figure 3:**
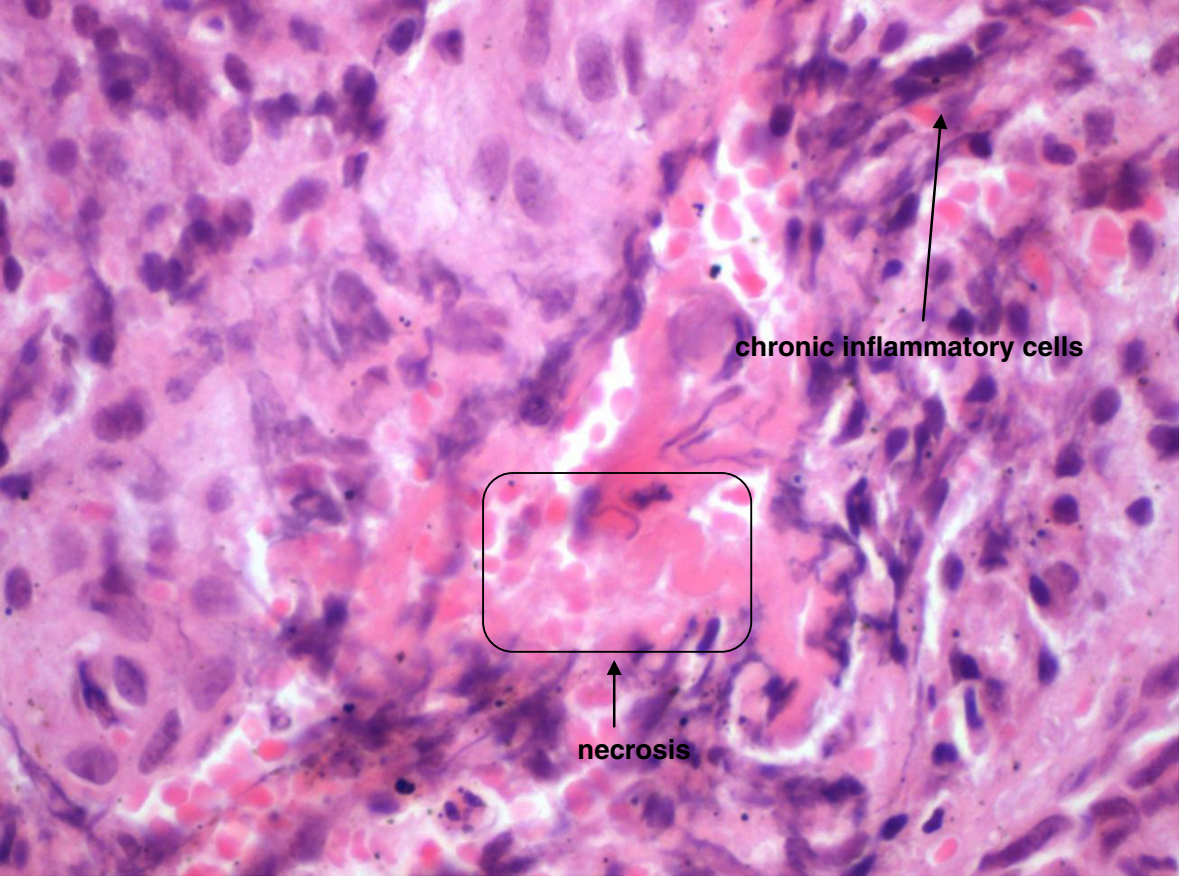
BRONJ: Chronic inflammatory cells infiltrate

**Figure 4:**
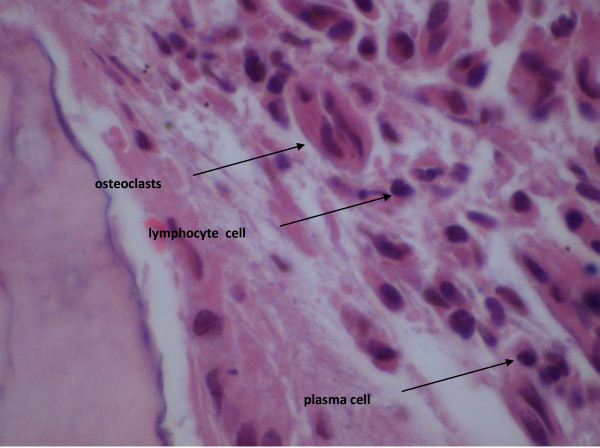
BRONJ: Osteoclasts, lymphocytes and plasma cells

The associated morphological features of this specific type of avascular necrosis are quite heterogeneous. Some areas of the affected bone with show extensive haemorrhage associated with massive chronic inflammatory cells infiltrate represented by lymphocytes and plasma cells which is best observed towards the surface of the bone (Figures 2, 3). In addition, a significant number of these cells are osteoclasts which continue to exert their characteristic bone resorptive action in parallel with the on-going lacunar depletion of osteocytes (Figure 4).

### BRONJ: the anti-angiogenic side effect of BPs treatment

It is known that angiogenesis or formation of new immature blood capillaries is an essential factor in healing of wounds as well as invasion of normal tissues by malignant cells [25]. Also, normal vascularization represents an essential requirement for tissue homeostasis, local immunity and adequate regeneration or repair of all vital tissues of the body, more so in case of bone due to its high turnover rate. In a recent study by Wehrhan et al. (2011), mucoperiosteal tissue samples from BRONJ cases and controls were assessed for vascularization with CD31 staining and neo-angiogenesis by CD105 evaluation. It was reported that although there was no difference in vascularization between sample groups, there were significantly fewer CD105-positive vessels in BRONJ samples suggesting that neo-angiogenesis was suppressed in BRONJ cases [26].

Vascular endothelial growth factor (VEGF), in circulation as well as local VEGF mRNA expression, is considered a classic parameter of angiogenesis. Remarkably, in non-small cell lung cancer cell lines, zoledronic acid promotes a significant reduction in mRNA and protein expression of VEGF [27]. Moreover, serum VEGF levels and other cytokines involved in angiogenesis such as interleukin-17, have also been found to decrease after administration of zoledronate or pamidronate [28-30]. It is very important to note that BPs determine an efficient decrease of VEGF at 24 hours after administration. The VEGF drop is sustained and it has been noted at 7 days after pamidronate infusion and at 21 days post zoledronate administration [28-30].

Although, there are no studies evaluating the duration of anti-VEGF effect after BPs treatment, it might be possible to be time limited. In vitro and in vivo experiments have revealed that clodronate is an efficient anti-angiogenic agent. The clodronate-related anti-angiogenesis seems to be related to its direct inhibitory action on endothelial cell proliferative activity coupled with inhibition of powerful pro-angiogenic factors such as fibroblast-growth factor 2 (FGF2) [31].

**Figure 5:**
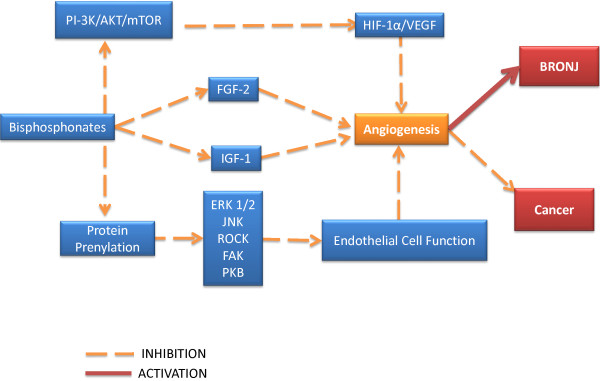
Bisphosphonates: mechanisms of anti-angiogenesis

Other authors have reported that NBPs have a significant anti-angiogenic effect inhibiting human umbilical vein endothelial cells (HUVEC) proliferation, adhesion, survival, migration and actin stress fiber formation by interfering with protein prenylation [32,33]. More importantly, these studies have revealed that NBPs such as zoledronic acid inhibit endothelial cells function and survival by acting on ERK1/2, JNK, Rock, FAK and PKB in a prenylation dependent reaction [32]. Recent studies performed on human breast cancer cell lines suggest that pamidronate and clodronate have a robust anti-angiogenic action on the HIF-1alpha/VEGF axis via inhibition of the PI-3K/AKT/mTOR signaling pathways. In addition, both agents inhibit the pro-angiogenic action of IGF-1 on breast cancer cells lines [34]. It is noteworthy to mention that the anti-angiogenic effect of BPs may be explained by their inhibition of the endothelial progenitor cell differentiation. Studies conducted in vitro have shown that at low doses, zoledronic acid inhibits the differentiation of endothelial progenitor cells while at high doses it will induce apoptosis of endothelial progenitor cells which seems to be achieved via inhibition of prenylation of small G proteins [35] (Figure 5).

Our own histological evaluation of patients treated with BPs has confirmed that BRONJ represents an avascular process of necrosis of the jaw bone due to the lack of blood vessels in the necrotic areas paradoxically associated with an on-going bone resorptive action induced by osteoclasts. However, numerous studies have revealed that bisphosphonates inhibit the osteoclasts by preventing their differentiation from their marrow precursors or inhibiting the mevalonate pathway which is crucial for their functionality [36,37]. Therefore, the presence of multinucleated giant cells osteoclasts in BRONJ patients is puzzling. As mentioned before, we have been able to observe routinely osteoclasts in histological slides prepared with tissue removed from the lesional and peri-lesional BRONJ area. This suggests that the anti- osteoclastic action of BPs could be limited and once they re-appear in the bone, the destruction is increased. More importantly, it is likely that the anti-osteoclastic and the anti- angiogenic action of BPs are not achieved through the same pathway but their final effect is summative in producing BRONJ.

Closely related to their anti-angiogenic effect, the BPs may affect the soft tissue structures seen in the immediate vicinity of the jaw or maxillary bone, potentially creating a vicious circle whereby the anti-angiogenesis is enhanced and complicated by soft tissue cells damage.

**Figure 6:**
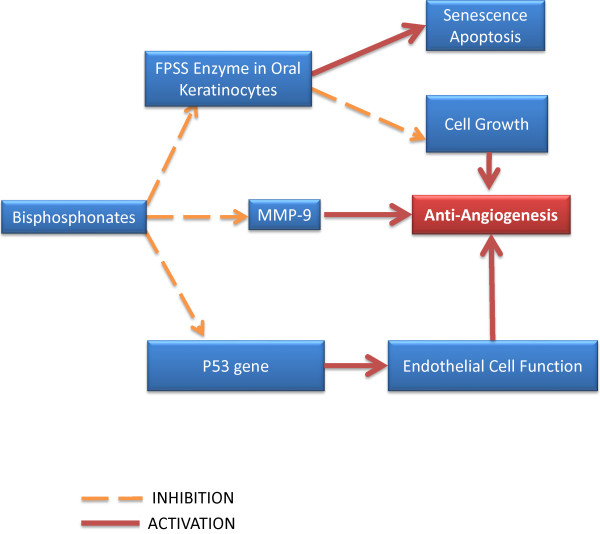
Bisphosphonate effects on oral keratinocytes

In studies with oral keratinocyte/model, BPs were shown to induce senescence, apoptosis and inhibition of cell growth by blocking FPPS enzyme of cholesterol biosynthetic pathway [38-43] which probably indirectly interfere with the blood supply of the bone. This is the same molecular mechanism through which NBPs inhibit osteoclast function, promoting apoptosis and preventing invasion of tumor cells (Figure 6).

A histological evaluation of BRONJ has revealed a reduced p53 gene expression of tissue samples via mevalonate pathway which results in a reduced numbers of basal epithelial progenitor cells which could result in an impaired healing capacity of oral mucosa [44]. An immunohistochemical study conducted on dogs has shown that MMP-9, a factor of paramount importance for the extracellular matrix homeostasis, is significantly decreased in subjects treated with zoledronic acid, suggesting that BPs promote epithelial cell apoptosis [45] (Figure 6). All of the above mentioned abnormalities may in fact be at work in BPs treated patients and they may act in concert to promote extensive bone destruction as seen in BRONJ.

However, recent studies have revealed that the reduced migration ability of various cell types of hard and soft tissues such as bisphosphonate-treated human umbilical cord vein endothelial cells (HUVEC), fibroblasts (normally a source of pro-angiogenic factors) and osteogenic cells may be potentially prevented by administration of factors with anti-BPs action. Some authors have claimed that the mevalonate pathway metabolite, geranylgeraniol (GGOH) or naturally occurring farnesol could an anti-BPs effect healing the lesions seen in BRONJ [46,47]. Therefore, these factors may represent the basis for a potential curative therapeutic protocol in BRONJ patients but more validation studies are necessary before a pilot study could be implemented [46,47].

Clearly bisphosphonates have a demonstrated multimodal ability to interfere with normal angiogenesis. Given the critical role of vasculature development in tissues and in particular bone, where the turnover rate is markedly higher than in other tissues, this represents a potential process that may therefore be intimately associated with the development of necrotic bone conditions such as that seen in the oral maxillofacial area of patients on high potency bisphosphonates following routine dental procedures.

This research is in compliance with the Helsinki Declaration. Approval has been obtained from Griffith University Human Research Ethics Committee. Written informed consent was obtained from the patient for publication of this report and any accompanying images.

## Competing interests

The authors declare that they have no competing interests.

## Authors’ contributions

DS: drafted part of the manuscript including the digital graphics, performed the literature search, assisted the histopathological selection, SI: designed the project, drafted part of the manuscript including the digital graphics, provided clinical feed-back, analysis and interpretation and supervised DS, MS: provided histopathological research information and evaluation, helped drafting the manuscript, SH: provided histopathological analysis, experimental research information and evaluation, helped drafting the manuscript, TSP: helped drafting the manuscript, provided research and clinical interpretation , performed digital imaging processing, KB: helped drafting the manuscript, provided basic research information and histopathological evaluation, EBP: drafted part of the manuscript, performed histopathological selection, processing and evaluation, experiential data interpretation, designed the project and supervised DS, RIM: designed the project, drafted the manuscript, provided clinical background and analysis, performed the literature search, evaluated experimental data and digital imaging processing. All authors read and approved the final manuscript.
